# Mature habitats associated with genetic divergence despite strong dispersal ability in an arthropod

**DOI:** 10.1186/1471-2148-7-52

**Published:** 2007-04-02

**Authors:** Seiji Ishida, Derek J Taylor

**Affiliations:** 1 Department of Biological Sciences, The State University of New York at Buffalo, Buffalo NY 14260, USA

## Abstract

**Background:**

Populations may be bound by contemporary gene flow, selective sweeps, and extinction-recolonization processes. Indeed, existing molecular estimates indicate that species with low levels of gene flow are rare. However, strong priority effects and local selective regimes may hinder gene flow (despite dispersal) sending populations on independent evolutionary trajectories. In this scenario (the monopolization hypothesis), population differentiation will increase with time and genealogical evidence should yield ample private haplotypes. Cyclical parthenogens (e.g. rotifers and cladocerans such as *Daphnia*) have an increased capacity for rapid local adaptation and priority effects because sexual reproduction is followed by multiple generations of clonal selection and massive egg bank formation. We aimed to better understand the history of population differentiation and ongoing gene flow in *Daphnia rosea *s.l., by comparing population and regional divergences in mature unglaciated areas and younger previously glaciated areas. We also examined the timing and paths of colonization of previously-glaciated areas to assess the dispersal limitations of *D. rosea *s.l. We used DNA sequence variation (84 populations and >400 individuals) at the mitochondrial ND2 and nuclear HSP90 loci from Holarctic populations for our genetic analyses.

**Results:**

The genetic evidence indicated pronounced historical structure. Holarctic mtDNA phylogenies of *D. rosea *s.l. revealed three geographically restricted and divergent clades: European, Siberian and Japanese/American. The Japanese/American clade showed marked population genetic structure (*F*_*ST *_> 0.8) that was weakly associated with geographic distance, and a high proportion of private haplotypes. Populations from older unglaciated habitats (i.e., Japan) showed higher DNA sequence divergences than populations from presumed younger habitats (i.e. non-Beringian North America) with nDNA and with mtDNA. Mismatch analyses of mtDNA and nDNA were consistent with a single rapid post-glacial expansion of *D. rosea *that covered most of the New World.

**Conclusion:**

Our evidence agrees with negligible gene flow after founding, and the accumulation of genetic divergence with habitat age. Existing direct evidence and our mismatch analyses indicate that the pronounced population differentiation is unlikely to be due to dispersal limitation. Instead, priority effects and local selection regimes may play a role in limiting gene flow. The results challenge the notion that lacustrine populations of cladocerans are generally unified by contemporary gene flow.

## Background

The Biological Species Concept assumes that gene flow limits population differentiation and acts as a cohesive process for species [1]. Related potential cohesive forces are selective sweeps of advantageous alleles, hybrid vigour involving immigrant alleles, and genetic rescue from frequent bouts of extinction and recolonization [2-4]. However, Ehrlich and Raven [2] argued that gene flow itself is rarely sufficient to bind populations of a species together as a unit of concerted evolution even in the face of strong dispersal. Instead of dispersal, natural selection is the primary force governing gene flow among populations. Although Ehrlich and Raven [2] recognized that selection may act as a cohesive force, they posited that in most cases selection keeps populations on independent evolutionary trajectories, and predicted that the increasing use of refined genetic tools would eventually reveal that gene flow is unimportant in unifying populations. Similarly, in their monopolization hypothesis, De Meester et al. [3] concluded that many aquatic populations possess weak ongoing gene flow – local adaptations, priority effects, and large egg/seed banks may restrict gene flow despite pronounced dispersal from nearby populations. Thus, the disruptive selection and monopolization schools view the population as the primary unit of evolution whereas the cohesion school views the species as the primary unit of evolution.

Despite the predictions by Ehrlich and Raven [2], modern gene flow studies have strongly favoured the primacy of cohesive gene flow over disruptive local selection [4,5]. Indeed, Slatkin [6] declared that he knows of no cases where molecular estimates of gene flow are low despite a high level of observed dispersal (i.e., direct estimates). Moreover, nearly all of the low molecular estimates of gene flow in animals appear inflated by historical processes (e.g. the inclusion of potentially vicariant phylogroups) [4]. So, Ehrlich & Raven's key prediction of marked local population genetic differentiation in the face of dispersal appears unsupported. An additional blow against disruption/monopolization hypotheses is the recent theoretical and empirical evidence that the main role of selection may be as an enhancer (not a disruptor) of gene flow via heterosis or universal selective sweeps [4,7].

Still, it is possible that the existing molecular estimates of gene flow, which are based largely on obligate sexual species, are unrepresentative of animals. De Meester et al. [3], for example, ranked cyclical parthenogens as the animal breeding system with the greatest potential for monopolization. Cyclical parthenogens (e.g. rotifers and cladocerans) have an increased capacity for rapid local adaptation over other breeding systems because of their frequent sexual reproduction followed by multiple generations of clonal selection [5] and persistent priority effects [8]. There is now considerable evidence for the rapid evolution of population-specific adaptations in cladocerans such as body size, defensive structures, phototactic behavior, resistance to algal toxins, and parasite resistance[7,10-12]. There is also growing direct evidence that these local adaptations arise in species with strong dispersal of propagules by avian vectors [3,9,10] and wind [11,12]. Colonization studies indicate that cyclical parthenogens such as daphniids appear to have strong dispersal capacity [13]. Additional evidence for the strong natural dispersal capacity of cladocerans comes from paleogenetic analysis of cladoceran embryos in aged African lake sediments [14]. A single asexual hybrid clone of North American *Daphnia pulex *appears to have been introduced into a central African lake and spread naturally throughout the continent in less than 60 years. Likewise, clones in the *Daphnia pulex *group appear to have dispersed naturally across immense distances in the Arctic [15]. Although nearly all cladocerans lack the potential heterotic advantage and asexual propagules of the *D. pulex *group, the examples do speak to the strong passive dispersal capacity of daphniids. Thus, cladocerans appear to be biologically predisposed to the scenario envisioned by Ehrlich and Raven where local selection disrupts populations despite dispersal.

Does the available indirect evidence then support low gene flow despite strong dispersal in cladocerans? There are many population genetic studies on cladocerans and most report moderate to strong population structure using relative estimates [3]. However, this result constitutes weak evidence for low gene flow because it is well known that founder effects and long-distance dispersal events (which both inflate *F*_*ST *_values) have been common in cladocerans [9,19-21]. Additional evidence is necessary to tease apart ongoing gene flow from monopolization. Analysis of population history offers one possible solution as the monopolization hypothesis and the ongoing gene flow hypothesis lead to different predictions about population genetic differentiation over time. Under ongoing gene flow, population differentiation should decrease with time since founding [8,16]. Moreover, gene flow and recombination should reduce allelic sequence divergence and the proportion of private alleles. But, with little or no ongoing gene flow, haplotype frequency differences, the proportion of private alleles, and allelic sequence divergences should increase among populations with time.

Existing population history studies of *Daphnia *do support the predictions of ongoing gene flow, but the generality of these results for the genus are uncertain. Potentially older populations from large lakes show less genetic differentiation (i.e. lower *F*_*ST*_'s) than populations from presumed younger habitats such as small ponds [22-28]. However, the higher turnover rates in ponds compared to large lakes may lead to relatively high *F*_*ST*_'s in ponds [17]. A detailed 17-year longitudinal genetic study of two *Daphnia *species with rock-pool subpopulations of known age showed a clear erosion of allozyme-based *F*_*ST*_'s with time [11]. Still, the rock pools also appear to lack the large effective egg banks and strong spatial variation in biotic selection pressures (e.g. fish versus no fish) that are characteristic of cladoceran populations from larger water bodies. Finally, a Holarctic analysis of the *Daphnia tenebrosa *s. l. using mtDNA RFLP's showed significant genetic structure among younger glaciated regions but no genetic structure among older non-glaciated regions. *D. tenebrosa *is a member of the *D. pulex *group that normally produces strictly asexually at higher latitudes, making it less susceptible to the proposed mechanisms of monopolization in sexual cladocerans [15].

Studies of sexual *Daphnia *that use the absolute estimates of population divergences yielded by DNA sequences are still uncommon (see Paland et al[18] for an asexual example). This absolute measure of population divergence will be sensitive to sampling error, but should be less inflated by founder effect. De Gelas and De Meester [19] provided evidence from COI mtDNA sequences that the present day population structure in European *Daphnia magna *is largely the result of a mosaic formed by historical recolonization from four glacial refugia rather than from ongoing gene flow. They detected some divergent lineages in refugial areas but lacked samples for a detailed population analysis of refugial regions. In the present study we obtained both relative and absolute estimates of population genetic divergence; used both nuclear and mtDNA genomic sequences; sampled populations from throughout the Holarctic range of the study species (including at a local scale to understand structure from long distance dispersal); and studied a sexual species of *Daphnia *that commonly occupies permanent, weakly-disturbed waters with a large range of habitat ages.

The study species, *Daphnia rosea *s.l. is a common component of oligotrophic permanent lakes and ponds in the boreal, alpine, and subarctic zones of the Holarctic [30-34]. Although habitat-specific head shapes are common in *D. rosea*, the most recent morphological treatments have supported one Holarctic species [20,21]. Allozyme and mtDNA phylogenetic analyses of a few specimens from the extremes of the geographic range reported a western European clade (*Daphnia rosea*) and a North American clade (*D. dentifera*) [22,23], but provided no evidence for more than one species in each of these allopatric clades. *D. rosea *s.l. hatch from resting eggs in the Spring and reproduce asexually for at least eight generations [11]. In the fall, some individuals produce sexual propagules that sink to the sediment and function as egg banks [24], but vast numbers of resting propagules may float forming a surface scum and shoreline deposit [25]. The direct evidence for strong dispersal, potential regional genetic structuring, cyclical parthenogenetic breeding system, rapid local adaptations [26] and frequent occupancy of permanent habitats suggests that *D. rosea *s.l. is a good candidate for testing the historical predictions of the monopolization hypothesis.

Here, we tested the hypothesis that *D. rosea *s.l. conforms to the population structure of a sexual vagile species, where ongoing gene flow is sufficient to offset population genetic divergence. We aimed to compare populations from regions that are potentially of different ages by sampling in well-established glacial refugia (Japan and Beringia) and in younger glaciated areas (temperate North America) [27]. We also assessed the timescale and routes of recolonization of glaciated areas to understand if evolutionary significant dispersal limitation exists in *D. rosea *s.l. We found evidence for marked historical structure and little evidence for ongoing gene flow. Populations from unglaciated regions exhibited significantly higher sequence divergences for nuclear and mitochondrial DNA compared to populations from glaciated North America. We found the genetic signature of a single rapid population expansion for most of the glaciated New World, suggesting that dispersal limitation is insignificant on an evolutionary timescale. The results are consistent with the monopolization hypothesis where priority effects and selection trump contemporary gene flow, rendering the population or local metapopulation as the units of evolution.

## Results

**Figure 1:**
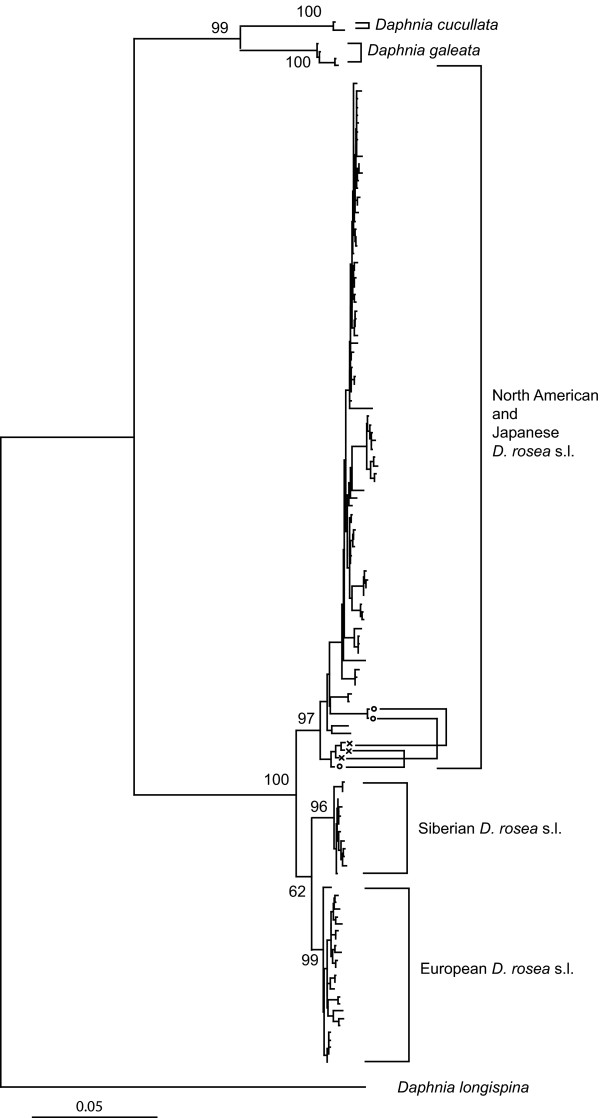
**Three regional phylogroups in Holarctic *Daphnia rosea *s.l**. Holarctic Neighbor-joining phylogeny based on mitochondrial ND2 sequences of *D. rosea *s.l. and its closely related species. Numbers on branches indicate bootstrap support. *D. rosea *s.l. has three geographical groups (Japanese/America, Siberia and Europe). Each individual of two Japanese populations (Kazefuki-o-ike and Hakuba-o-ike, Nagano) had two copies of sequences (connected with a line): one (cross) was a putative pseudogene, which had one stop codon; and stop codons and frameshifts where absent from the other (circle).

**Figure 2:**
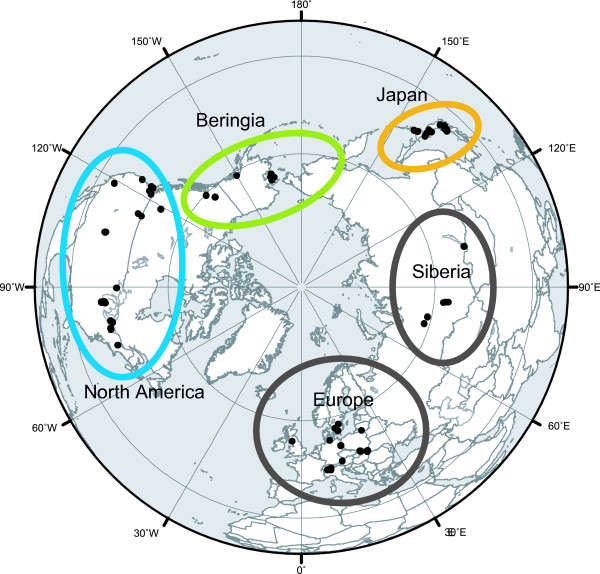
**Holarctic map showing populations and regions studied for *Daphnia rosea***. Holarctic map of the 84 sampling locations of *D. rosea *s.l. Japan (orange) and Beringia (green) represent possible refugial areas while North America (blue) indicates a potentially younger region. Siberian and European populations (gray) were not used for phylogenetic analysis only because of reduced samples and less information about putative refugia.

We found marked regional structure of *D. rosea *s.l with three divergent allopatric mtDNA clades: European; Siberian and Japanese-American and no indication of recent long-distance dispersal among regions (Fig. 1 and 2) [see Additional file 1]. The ND2 alignment was 937 bp with 122 unique ingroup haplotypes, no indels (insertions or deletions), and 421 variable sites. The best-fit ML model was TIM + I + G (transition model with invariable sites plus gamma distribution). The ML distance between the European and Siberian clades was 4.47%, while the ML distances from the American-Japanese clade to the European clade and the Siberian clade were 7.61% and 8.13% respectively. We suspected the existence of a pseudogene in two Japanese populations (Kazefuki-o-ike and Hakuba-o-ike in Nagano) because of ambiguous sequences, and cloned PCR products to further examine the possibility. Cloning of the PCR products revealed that each individual of the populations had two sets of haplotypes (Fig. 1); one with a complete reading frame and one with stop codons at 127–129 bp. Ambiguous sites and stop codons were absent from the mtDNA of all individuals sampled outside of these two populations.

**Figure 3:**
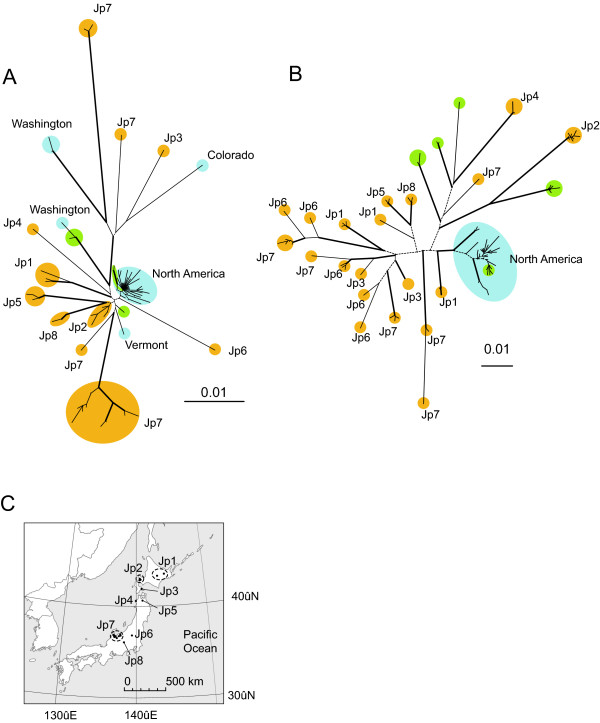
**Greater absolute genetic divergences and regional divergences in unglaciated regions compared to glaciated regions for *Daphnia rosea***. Unrooted Neighbor-joining phylograms of mitochondrial ND2 gene sequences (A) and nuclear HSP90 gene sequences (B) for Japanese and North American *D. rosea *s.l. The HSP90 sequences are from a subset of specimens represented on the ND2 tree. Colors represent geographic location: blue (North America except for Alaska, USA and Yukon, Canada); green (Beringian North America, i.e. Alaska and Yukon); and gold (Japan). Thick branches have more than 70% bootstrap support, and dot branches have less than 50% bootstrap support. The map (C) shows Japanese regions (Jp1-8) and populations. Note the lack of sharing of nuclear and mitochondrial haplotypes among Japanese regions (Jp1-8).

**Figure 4:**
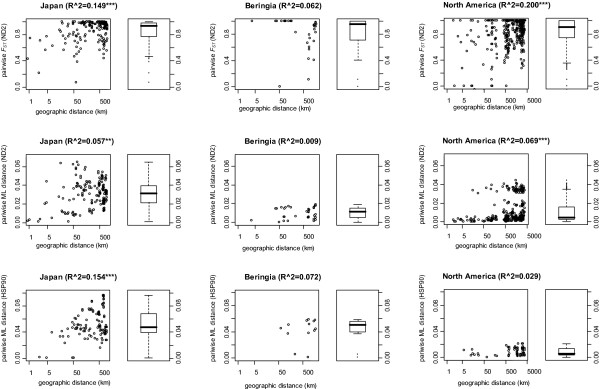
**Summaries of relative and absolute pairwise population differentiation and their associations with geographic distance in *Daphnia rosea***. Row one: associations between pairwise *F*_*ST*_'s (from ND2 sequences) and geographic distances (log); Row two: associations between pairwise ML distances (from ND2 sequences) and geographic distances (log); and Row three: the associations between pairwise ML distances (from HSP90 sequences) and geographic distances (log). Population-level information for HSP90 *F*_*ST *_estimates was unavailable. Boxplot summaries of the pairwise genetic comparisons are shown to the right of each scatterplot. Regression coefficients are given above each scatterplot (*** – P < 0.001; ** – P < 0.01). The first two columns are from presumed glacial refugial regions of *Daphnia rosea *and the last column is from glaciated North America.

We found extremely high estimates of relative population structure within both glaciated and non-glaciated regions of the North American – Japanese mtDNA clade; for non-Beringian North America *F*_ST _= 0.936 (N*m *= 0.02) and S_nn _= 0.744 (P < 0.001); for Beringia *F*_ST _= 0.869 (N*m *= 0.04) and S_nn _= 0.751 (P < 0.001) and for Japan *F*_ST _= 0.804 (N*m *= 0.09) and S_nn _= 0.834 (P < 0.001). Likewise, mtDNA haplotype diversities were high for each region: 0.939 ± 0.017 (95% CI) for non-Beringian North America, 0.869 ± 0.017 for Beringia, and 0.963 ± 0.006 for Japan. Private mtDNA haplotypes (which occur in only one population) were common: 94% of the 48 North American haplotypes and 91% of 34 Japanese haplotypes. Population structure among local sites (within 100 km distance) was also extremely pronounced; *F*_*ST *_= 1.000 in western Alaska, USA (*N *= 5); *F*_*ST *_= 0.988 in Colorado, USA (*N *= 3); *F*_*ST *_= 0.935 in Michigan, USA (*N *= 3); *F*_*ST *_= 0.916 in Ontario, Canada (*N *= 3); *F*_*ST *_= 0.833 in New York, USA (*N *= 3); *F*_*ST *_= 0.645 in central Honshu, Japan (*N *= 8, Jp-7 in Fig. 3C); *F*_*ST *_= 0.917 in western Hokkaido, Japan (*N *= 3, Jp-2 in Fig. 3C) and *F*_*ST *_= 0.915 in eastern Hokkaido, Japan (*N *= 2, Jp-1 in Fig. 3C). For Japan, AMOVA revealed that 55.84% of the variation in ND2 sequences occurred among regions, 34.62% of the variation occurred among populations within a region and 9.54% of the variation occurred within populations. Although geographic distance among populations explained a significant amount of the variation in pairwise *F*_*ST*_*'s *in Japan and in non-glaciated North America, the amount of variation explained was low (Fig. 4; 6–20%).

Unlike the universally high relative measures, absolute measures of population divergences populations from presumed older habitats (i.e. Japan and Beringia) were generally greater than the divergences among populations from glaciated habitats (i.e. non-Beringian North America) in mtDNA and in nDNA sequences (Fig. 3). The ND2 alignment of the Japanese-American clade contained 82 unique haplotypes (937 bp), no indels, and 199 variable sites. The best-fit ML model was TrN + G (Tamura – Nei with gamma distribution). Average pairwise ML distances (using the best fit model and parameters) among populations were much higher in Japan (2.84% ± 0.14% in 95% CI) than in non-Beringian North America (1.01% ± 0.08%) and Beringian North America (0.88% ± 0.18%). mtDNA nucleotide diversities were also greater in Japan (0.0234 ± 0.0026) than in non-Beringia (0.0104 ± 0.0020) and in Beringia (0.0088 ± 0.0010).

The HSP90 tree consisted of a subset of specimens represented on the ND2 tree of the Japanese-American clade. The total alignment was total 784 bp with 192 variable sites and 95 unique alleles. Two putative introns were detected: Intron I (69 bp) between 87 bp position and 155 bp position; and Intron II (69 bp) between 448 bp position and 516 bp position. No indels were found in exons. 2–5 bp and 3–7 bp indels were found in Intron I and Intron II, respectively. The best-fit ML model was GTR + I + G (General time-reversible model with invariable site plus gamma distribution). Average pairwise ML distances (using the best-fit model and parameters) among populations (nDNA) were much higher in nonglaciated regions (Japan 5.04% ± 0.14%; Beringia 4.15% ± 0.37%) than in non-glaciated North America (1.07% ± 0.06%).

**Figure 5:**
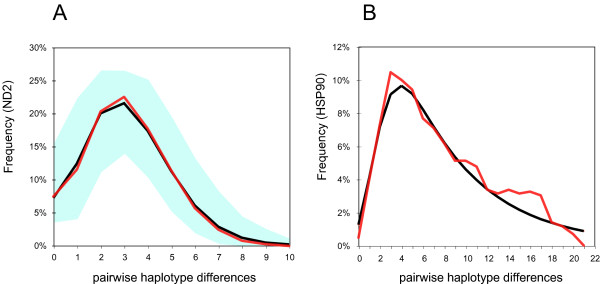
**Expansion patterns in mismatch distributions for temperate North American populations of *Daphnia rosea***. The distribution of pairwise haplotype differences (number of differences) within the major mitochondrial clade (A) and the major nuclear clade (B) of North American populations. Black line indicates the observed distribution. Red line indicates the expected distribution based on the spatial expansion model. Light blue zone indicates the 95% CI range of expected distribution based on the spatial expansion model. The nuclear distribution lacks the 95% CI range, because the least-square fitting algorithm in ARLEQUIN failed to converge.

North American populations showed genetic signatures of a bottleneck and postglacial expansion. Most North American haplotypes were restricted to a star-like clade of closely related haplotypes (Fig. 3A) [see Additional file 2]. The clade had significant negative values of neutrality tests (*p *< 0.001 in Tajima's *D *and Fu's *F*_*S*_). Still, just two mitochondrial haplotypes were shared among populations in the star-like North American clade. One of these shared haplotypes formed the central haplotype in a haplotype network [see Additional file 2] and was widely distributed in North America: one population in Alaska (USA), one population in Yukon (Western Canada), one population in Wisconsin (USA), three populations in Michigan (USA) and two populations in Indiana (USA). The other shared haplotype was detected only within a 120 km diameter region near the Michigan – Indiana border. Pairwise differences of all sequences (n = 199) in the clade formed a unimodal distribution pattern, which fit the expected distributions of spatial expansion (Fig 5A; *P*_[simulated SSD > observed SSD] _= 0.93) and pure demographic expansion (*P *= 0.38). The parameter Tau in the spatial expansion model was 3.228 (1.582–4.288 in 95 % CI). Pairwise differences of the non-Beringian sequences (n = 175) in the clade formed a unimodal pattern, which fit the expected distributions of spatial expansion (*P *= 0.45) but not of pure demographic expansion (*P *< 0.05). The parameter Tau in the spatial expansion model was 3.088 (1.544–4.080 in 95 % CI).

Similar to the mtDNA, we detected a clade comprised of the non-Beringian North American populations and two Yukon populations with nDNA (Fig. 3B) [see Additional file 3]. The clade had significant negative values of neutrality tests (*p *< 0.05 in Tajima's *D *and *p *< 0.001 in Fu's *F*_*S*_). Three nuclear haplotypes were detected as shared among populations in this North American clade. Pairwise differences of all sequences (n = 43) in the clade were distributed in a unimodal pattern, which is similar to the expected distributions under models of spatial and sudden expansions (Fig 5B). The parameter Tau in the spatial expansion model was 2.427. However, the least-square fitting algorithm in ARLEQUIN [28] did not converge, suggesting that the nuclear data poorly fit the available models. Thus, the *P*-value for the available models and the 95% CI of the expected distributions were not available. Similarly, pairwise differences of the non-Beringian nuclear sequences (n = 35) were distributed in a unimodal pattern, and the least-square fitting algorithm did not converge.

## Discussion

The phylogeographic and population genetic results are consistent with the monopolization hypothesis where gene flow is insufficient to prevent population divergence [2,3]. *Daphnia rosea *s.l. may represent Slatkin's [6] missing gene flow category with strong estimated population structure despite strong dispersal abilities. Morjan & Rieseberg's [4] review provides an indication of how extreme the population structuring is for *D. rosea*. They listed a mean *F*_*ST *_in animals of 0.45 (150 species) for mtDNA and 0.2 (781 species) for nuclear markers. A small percentage of studies (24 animal species) had a population subdivision estimate of > 0.8. However, in each of these cases, the estimate was calculated across divergent geographic clades, suggesting that historical relationships inflated the estimate. Our finding of *F*_*ST *_> 0.8 subdivision among populations within a regional clade and genetically divergent haplotypes among Japanese populations reveals that the vagile *D. rosea *s.l. also possesses among the highest estimates of population subdivision yet recorded for animals.

Unlike previous studies, and contrary to the predictions of ongoing gene flow, we found by two crude measures of habitat maturity that older populations possessed as much or more genetic structure than presumed younger populations. Our results indicate that populations of *Daphnia *from large lakes and large ponds can possess strong structure – stronger than has been reported from small pond species [17,29]. Part of the difference between our estimates and prior studies arises from the increased mutation rates of our markers. But this cannot be the only explanation as some studies of pond cladocerans [29,30] have used rapidly evolving markers (i.e. control region of mtDNA and microsatellites). Nor is the pattern restricted to the present study species as Ishida & Taylor [31] have recently found similarly strong population structure using the same markers in a large study of the lacustrine specialist, *Daphnia galeata*. The absolute measures of genetic divergence from presumed older habitats or metapopulations in unglaciated areas (Japan and Alaska) were generally much greater than those population genetic divergences among younger habitats (glaciated areas). The contrast between glaciated and unglaciated areas was apparent in both the mitochondrial and the nuclear genomes. Only the Beringian – North American contrast for ND2 distances revealed similar levels of structure. Analysis of more populations from central Beringia is necessary to determine if the contrast is real. Both the increased divergence with age of habitat and the very high proportion of private haplotypes found in most populations are inconsistent with ongoing gene flow acting to homogenize populations. However, they do satisfy a major prediction of the monopolization hypothesis: increased differentiation and divergence with population age.

The roles of local selection, ongoing gene flow with drift, and dispersal limitation are difficult to tease apart as contributors to the observed population structuring of *D. rosea*. Ongoing gene flow with drift probably occurs, but it seems unlikely given the lack of allele sharing and relatively high sequence divergences among geographically close regions in Japan with both nDNA and mtDNA (see also De Gelas and De Meester [19]). Colonization studies [11,13], the massive production of resting propagules, and the evidence for rapid postglacial colonization of vast areas of the northern hemisphere indicate that dispersal limitation may also be weak. The population genetic signature of *D. rosea *s.l. in North America is consistent with postglacial expansion from a Beringian refugium. A severe genetic bottleneck in glaciated North America was clear from both the nuclear and the mitochondrial results. Diverse nuclear lineages in the Northern Pacific Coast (especially Alaska) and reduced genetic diversity in glaciated regions indicates population expansion from Beringia. The mitochondrial North American clade consistently showed the spatial expansion patterns in mismatch distributions with or without Alaska and Yukon populations. The spatial expansion model assumes that subdivided populations expand the distribution range and increase the total number of the individuals, while a pure demographic expansion model assumes that populations are not subdivided. The observed high value of *F*_*ST *_and the high frequency of private haplotypes among the Am1 populations [see Additional file 1] indicate subdivided populations. The star-like haplotype network [see Additional file 1] and the significant negative values of neutrality tests were also consistent with the expansion patterns of this clade. Using *Tau *= 2ut and assuming a divergence rate of 2.0% per million years for arthropod mitochondrial protein-coding genes [32], the expansion time is estimated to be 86 000 years ago (42 000–113 000 years ago in 95% CI) for populations including Alaska and Yukon, and 82 000 years ago (41 000–109 000 years ago in 95% CI) for populations excluding Alaska and Yukon. However, the relationship between the age and the evolutionary rate can be described by a vertically translated exponential decay curve, where the short-term (< 1–2 Myr) mutation rate gets exponentially higher, while the long-term (> 10 Myr) substitution rate is constant. The estimated expansion times could be overestimated more than 10 times because of the recent events (< 0.1 Myr B.P.) [33]. Thus, the spatial expansion of *D. rosea *s.l. in North America might have occurred rapidly after the last Ice Age (< 15 000 y B.P.). As both Japan and Alaska contain ample migratory waterfowl vectors, and our results provide evidence that much of the North American continent was rapidly colonized after glaciation, there are no reasons to believe that dispersal differences or limitations account for the differing divergence patterns between Japan and North America. Also, the refugial areas represent relatively small geographic areas (e.g. Japan) compared to the vast glaciated regions (e.g., much of North America), so the divergence patterns are not attributable solely to isolation by distance.

Of course, indirect estimates of gene flow apply only to the loci under study. Selective sweeps of globally favourable alleles may occur at unsampled loci [4]. We note that the inclusion of mtDNA in our study makes our conclusion of strong population differentiation conservative – mtDNA is perhaps one of the most susceptible loci to homogenizing selective sweeps in animals [34]. We also note that during the multiple generations of clonal reproduction in *D. rosea*, mtDNA and nuclear genomes are linked, increasing the potential for background selection against immigrant genes [3,29]. Our finding of generally concordant divergence patterns for nuclear and mitochondrial loci and the frequent observation of stable, lake-specific body shapes in *D. rosea *s.l. [20] suggests that the strong population differentiation is not merely a locus-specific phenomenon.

If populations of *Daphnia rosea *are indeed exposed to negligible gene flow despite dispersal, then what are the evolutionary consequences? Speciation by habitat isolation or vicariance may occur if populations are separated in distant glacial refugia. Most populations of *D. rosea *have likely gone extinct from the regular waves of catastrophic Pleistocene glaciation or experienced turnover as local selection regimes changed. Indeed, the shape of the mtDNA tree for *D. rosea *is consistent with the recolonization of the Holarctic from at least three geographically – distant bottlenecked glacial refugia. Our evidence that local regions continue to diverge over time (perhaps over hundreds to thousands of years) in vagile *D. rosea*, suggests that the evolutionary legacy of monopolization is perhaps the stabilization of countless innovations and adaptations in local metapopulations. Some of these local adaptations such as parasite resistance [26] and defensive structures on the head [20] have already been identified for *D. rosea*.

Although we present a case of marked population differentiation and sequence divergence in cladocerans, it is difficult to estimate how taxonomically widespread monopolization is. However, if a mixed breeding system and differing local selection regimes are important drivers of monopolization, then the patterns we found in *D. rosea *s.l. are perhaps more widespread among eukaryotes. More work is necessary to understand the causes of monopolization. Future genetic analysis of preserved egg/embryo banks of cladocerans in lake sediments and permafrost may offer detailed window into the process.

## Conclusion

Our findings are consistent with negligible gene flow after founding, and the accumulation of genetic divergence with regional age. *Daphnia rosea *s.l. showed increased levels of population divergence in mature unglaciated regions compared to presumed younger glaciated regions. Previous direct evidence of vagility in *D. rosea *s. l. and our evidence for a rapid population expansion at the continental scale, suggests that most population differentiation is unlikely to be due to dispersal limitation. Instead, priority effects and selection may play roles in limiting gene flow. The results challenge the notion that sexual populations of lacustrine cladocerans are generally unified by contemporary gene flow.

## Methods

### Sample collection

Specimens of *D. rosea *s.l. were collected from 84 Holarctic locations [see Additional file 4]. As previous studies showed that *D. cucullata and D. longispina *were closely related species to *D. rosea *s.l. in the mitochondrial ND2 phylogeny [35], we used these species as outgroups [see Additional file 4]. All samples were preserved in absolute ethanol at room temperature, or frozen at -80°C. These specimens were morphologically identified according to Ueno [36], Brooks [20], Glagolev [37], Taylor et al. [22] and Flössner [21].

### DNA extraction, PCR and sequencing

Total genomic DNA was extracted by using QuickExtract (Epicentre). Samples were homogenized in 30–50 μL of the QuickExtract solution, incubated at 65°C for 2 h and 98°C for 10 min, and stored at -20°C. A *c*. 1000 bp fragment of the mitochondrial protein-coding NADH-2 (ND2) gene was amplified using the primers MetF3 (5'-GTT CAT GCC CCA TTT ATA GGT TA-3') and TrpR (5'-GAA GGT TTT TAG TTT AGT TAA CTT AAA ATT CT-3') [35]. A *c*. 760 bp fragment of the nuclear protein-coding HSP90 gene was amplified using the primers (5'-TTA CGA GTC CAG ATG GGC TT-3') and (5'-ATC CGT TAT GAA TCC CTG ACT GA-3') [38]. Each 50 μL PCR reaction consisted of 5 μL of extracted DNA, 10× PCR buffer [50 mM KCl, 1.5 mg MgCl2, 10 mM Tris-HCl pH 8.3, 0.01% (w/v) gelatin], 2 mM of each dNTP, 1 μM of each primer and 1 unit of *Taq *DNA polymerase. The PCR temperature profile for the mitochondrial ND2 gene was as follows: 40 cycles of 94°C for 30 s, 48°C for 30 s and 72°C for 1 min, and final extension at 72°C for 5 min. The PCR temperature profile for the nuclear HSP90 gene was as follows: 40 cycles of 94°C for 30 s, 50°C for 30 s and 72°C for 1 min, and final extension at 72°C for 5 min. Because ND2 PCR products from two Japanese populations had multiple peaks in their sequences, cloning was performed for the products using the TOPO TA Cloning Kit (Invitrogen). Then, three cultured colonies were used for sequencing. For HSP90, all PCR products were cloned using the TOPO TA Cloning Kit, and then four or five cultured colonies were sequenced. Sequences of ND2 and HSP90 were obtained in both directions by Genaissance pharmaceuticals (Connecticut, USA) or Roswell Park Cancer Institute (New York, USA). Sequences were assembled, edited with Sequencher 4.2 (Gene Code Corporation), and aligned manually with Se-Al 2.0 [39].

### Genetic analyses

For the mitochondrial phylogeny of Holarctic *D. rosea *s.l., we used a total of 403 individuals of *D. rosea *s.l from 84 Holarctic locations: 212 individuals from 30 non-Beringia North American populations (in North America except Alaska and Yukon), 43 individuals from nine Beringia North American populations (in Alaska, USA and Yukon, Canada), 97 individuals from 19 Japanese populations, 25 individuals from six Siberian populations, and 26 individuals from 20 European populations [see Additional file 4]. The best-fit maximum likelihood (ML) model was selected by hierarchical likelihood ratio tests in the program Modeltest 3.7 [40]. The ML distance was based on the best fit model and calculated using PAUP v4.0b10 [41]. An Neighbor-joining (NJ) tree was constructed with the ML distance and 1000 bootstrap replicates using PAUP.

Population level information was available for the mtDNA of specimens from North America and Japan. We calculated *F*_ST _with the estimator of Hudson et al. [42] and the nearest-neighbor statistic (S_nn_) of Hudson [43] as implemented in DnaSP 4.10 [44]. With Hudson et al.'s estimator of *F*_ST_, each polymorphic site in an alignment is treated as a locus and *F*_ST _= 1-*H*_w_/*H*_b_. *H*_w _is the mean number of sequence differences among different haplotypes sampled from the same subpopulation and *H*_b _is the mean number of sequence differences among samples from different subpopulations [42]. This estimator is equivalent to the *N*_*ST *_estimator of Lynch & Crease [45] without a Jukes-Cantor correction for multiple substitutions. S_nn _is a measure of how often the most closely related sequences (nearest-neighbors) occur in the same locality (water bodies in the present case). The number of private haplotypes (which occur in only one population) was counted. Haplotype diversities and nucleotide diversities were calculated using DnaSP [44].

We further analyzed phylogeography of the North American and Japanese populations using mitochondrial and nuclear data. For mitochondrial analysis, we used the same data of ND2 sequences that was used in construction of the Holarctic *D. rosea *s.l. phylogeny. For nuclear analysis, we used 36 individuals of the *D. rosea *s.l: 13 individuals from 13 non-Beringia North American populations, six individuals from six Beringia North America, and 17 individuals from 17 Japanese populations. Intron boundaries of the HSP90 sequences were identified by comparing arthropod HSP90 mRNA sequences (e.g. AY528900, AY423488) and by examining the intron-splicing signature sequences. Two introns were identified and aligned using clustalW. For mitochondrial and nuclear data of North American and Japanese *D. rosea *s.l., the best-fit ML model was selected by hierarchical likelihood ratio tests using Modeltest. ML distance was calculated using PAUP. NJ phylogeny was constructed with ML distance and 1000 bootstrap replicates using PAUP. The Mantel test [46] was used to determine the relationship between genetic distance (pairwise *F*_ST _from Arlequin 3.01 [28], and pairwise ML divergence) and geographic distance to test an isolation-by-distance model. The geographic distance was calculated using the great-circle distance formula. The Mantel test was implemented in IBD 1.52 [47] using 1000 permutations. The computer program TCS 1.21 [48] was used to estimate the mitochondrial haplotype network that illustrates all connections that have 95% probability of being the most parsimonious. Analysis of molecular variance (AMOVA) was carried out for Japanese regional groups (Jp1-8 in Fig. 3C) using the TrN + G (Tamura-Nei distance with gamma distribution) by Arlequin with 1000 permutations.

To address the population expansion of major North American clades in mitochondrial and nuclear phylogenies, we performed the neutrality tests of Fu's *F*_*S *_[49] and Tajima's *D *[50] and revealed the frequency distribution of the number of pairwise sequence differences (i.e. the mismatch distribution). The values of Fu's *F*_*S *_and Tajima's *D *are expected to have significantly negative values for population expansion (other explanations are background selection and hitch-hiking associated with selective sweeps). The significance of Fu's *F*_*S *_and Tajima's *D *were tested by random permutation using 1000 replicates in ARLEQUIN 3.01 [28]. The mismatch distributions were calculated using ARLEQUIN. The observed distributions were compared with the simulated distributions under the models of pure demographic expansion and spatial expansion. The model of pure demographic expansion assumes that non-subdivided populations suddenly expand in population size and increase the total number of individuals [51]. The model of spatial expansion assumes that subdivided populations expand the distribution range and increase the total number of individuals [52,53]. Both models estimate Tau = 2ut where u = m_T_μ. t is the estimated time of expansion, m_T _is the number of nucleotide sequences under study, and μ is the mutation rate per time. A least square procedure and bootstrap approach (1000 replications) provided the probability that the sum of squared deviations is lower in the observed distribution than in the simulated distributions, and calculated the 95% confidence intervals of the expected distribution and Tau.

## Authors' contributions

SI and DJT conceived and designed the research. SI performed the experiments and analyzed the data. SI and DJT wrote the paper. All authors read and approved the final manuscript.

## Supplementary Material

Additional file 1 **Neighbor-joining phylogeny based on mitochondrial ND2 sequences of *D. rosea *s.l. and its closely related species**. Numbers on branches indicate bootstrap support. Click here for file

Additional file 2 **Mitochondrial haplotype network of North American and Japanese clades**. Haplotypes are coloured according to geographical regions: blue (North America), green (Beringia), and red (Japan). White dots represent inferred haplotypes. The dot size is proportional to the number of different populations with the identical haplotype. Two divergent lineages in North America and several divergent lineages in Japan were excluded, because the computer program TCS failed to connect them. Click here for file

Additional file 3 **Neighbor-joining phylogeny based on nuclear HSP sequences of Japanese and North American *D. rosea *s.l**. Numbers on branches indicate bootstrap support. Click here for file

Additional file 4 **Lists of *Daphnia *specimens used in this study**. The lists provide the information of sampling locations, geographical position, IDs of ND2 and HSP90 sequences, number of individuals analyzed for ND2 sequences, and number of cloned colonies analyzed for HSP90 sequences. Click here for file
